# Cross-modal reaction of auditory and visual cortices after long-term bilateral hearing deprivation in the rat

**DOI:** 10.1007/s00429-019-01991-w

**Published:** 2019-11-28

**Authors:** M. Pernia, I. Díaz, A. C. Colmenárez-Raga, C. Rivadulla, J. Cudeiro, I. Plaza, M. A. Merchán

**Affiliations:** 1 Instituto de Neurociencias of Castilla y León-INCyL, Universidad de Salamanca, Salamanca, Spain; 2 Centro de Investigaciones Científicas Avanzadas (CICA), Facultad de Ciencias de la Salud, Universidad de A Coruña and Instituto de Investigaciones Biomédicas de A Coruña (INIBIC), A Coruña, Spain

**Keywords:** Visual evoked potentials, RT-qPCR, Quantitative immunocytochemistry, GluA2/3 (RRID: AB_90710), GAD 67 (RRID: AB_2278725), Parvalbumin (AB_10000344)

## Abstract

Visual cortex (VC) over-activation analysed by evoked responses has been demonstrated in congenital deafness and after long-term acquired hearing loss in humans. However, permanent hearing deprivation has not yet been explored in animal models. Thus, the present study aimed to examine functional and molecular changes underlying the visual and auditory cross-modal reaction. For such purpose, we analysed cortical visual evoked potentials (VEPs) and the gene expression (RT-qPCR) of a set of markers for neuronal activation (c-Fos) and activity-dependent homeostatic compensation (Arc/Arg3.1). To determine the state of excitation and inhibition, we performed RT-qPCR and quantitative immunocytochemistry for excitatory (receptor subunits GluA2/3) and inhibitory (GABAA-α1, GABAB-R2, GAD65/67 and parvalbumin-PV) markers. VC over-activation was demonstrated by a significant increase in VEPs wave N1 and by up-regulation of the activity-dependent early genes c-Fos and Arc/Arg3.1 (thus confirming, by RT-qPCR, our previously published immunocytochemical results). GluA2 gene and protein expression were significantly increased in the auditory cortex (AC), particularly in layers 2/3 pyramidal neurons, but inhibitory markers (GAD65/67 and PV-GABA interneurons) were also significantly upregulated in the AC, indicating a concurrent increase in inhibition. Therefore, after permanent hearing loss in the rat, the VC is not only over-activated but also potentially balanced by homeostatic regulation, while excitatory and inhibitory markers remain imbalanced in the AC, most likely resulting from changes in horizontal intermodal regulation.

## Introduction

To build the perceptual scene, the mammalian brain has developed neural circuits, specialised in analysing and mixing different sources of sensory information (Teichert and Bolz 2018). This ability requires a dynamic multimodal exchange of information along all stations of the sensory pathways, from the brainstem to the cerebral cortex. After optogenetic stimulation of auditory axons in mice brain slices, recordings from visual cortex (VC) pyramidal neurons and interneurons show increased EPSC responses, demonstrating the combined effect of excitation and inhibition underlying intermodal horizontal interactions (Ibrahim et al. 2016).

When one sensory system fails, the brain cortex reorganises its neural network to preserve intermodal processing, which is known as cross-modal plastic reaction. In deafened ferrets, de novo emergent somatosensory responses have been shown by single units recorded in the auditory cortex (AC), undoubtedly demonstrating a multimodal sensory conversion in the brain cortex after sensory deprivation (Allman et al. 2009). Since receptive fields result from inhibitory GABA interactions, demonstrated by iontophoresis (Firzlaff and Schuller 2001; Tremere et al. 2001), sensory conversion may reflect imbalanced multimodal neurotransmission between cortices.

In deaf mice, a potential increase in VC activation has been demonstrated by c-Fos overexpression (Teichert and Bolz 2017). In addition, after bilateral long-term deafness, c-Fos and Arc/Arg3.1 expression is significantly increased in immunoreactive neurons in the VC (Pernia et al., 2017). Thus, these data indicate that hearing deprivation induces long-term bimodal adaptive reorganisation of cortical neuronal networks and altered functional interactions between the VC and AC. Visual evoked potentials recordings (VEPs) is a fairly simple technique which provides reliable information on visual cortex activation, which has long been used in diagnostics (Hudnell et al. 1990). In humans, VC over-activation after permanent and long-term deafness has been demonstrated by VEPs (Neville et al. 1983; Campbell and Sharma 2014).

Cross-modal cortical interactions after deafness have been analysed from different points of view in animal models (Finney et al. 2001; Bizley et al. 2007; Allman et al. 2009; Meredith and Lomber 2011; Kok et al. 2014; among others); however, little evidence is available on how sensory cortices react after long-term deafness in adults. Thus, this study aimed to explore very long-term VC over-activation by long latency cortical VEP recordings under flash-light stimulation in a rat model of bilateral auditory deprivation; the model was also used to evaluate neuronal activation by analysing the gene expression of the activity-dependent early gene c-Fos.

In the brain cortex, activity-dependent synaptic plasticity is triggered after changes in synaptic strength of cortical neuronal networks induced by homeostatic regulation (Turrigiano et al. 1998—for review see Pérez-Otaño and Ehlers 2005). Consequently, imbalances between cortical excitation and inhibition, as expected after permanent auditory deprivation, may induce changes in neurotransmission and neuronal regulation in the sensory cortex. Therefore, this study also aimed to perform analysis of a well-characterised homeostatic plasticity regulator (Arc/Arg3.1), and neurotransmission markers for excitation (GluA2/3 AMPA R subunits) and inhibition (Gad65 and 67, GABA receptor subunits and parvalbumin) in the AC and VC.

## Materials and methods

This study was conducted according to Spanish (Royal Decree 53/2013–Law 32/2007) and European Union (Directive 2010/63/EU) guidelines for the care and use of laboratory animals. Protocols were approved by the Ethics Committee on Animal Experimentation of the University of Salamanca (Permit Number: 2012-265). Surgery was performed under monitored anaesthesia and all precautions were taken to minimise suffering. In this study, a total of 37, 3-month-old, Wistar rats weighing 250–300 g were used.

### Experimental groups

Group 1: VEP recordings. Control group *n* = 3; bilateral deaf animals *n* = 6.

Group 2: RT-qPCR to analyse the gene expression of *GluA2* AMPA R subunit, *Gad65, Gad67, Parvalbumin, Gabra1* and *Gabbr2*, and early genes *c*-*Fos* and *Arc/Arg3.1*. Control group *n* = 6; bilateral deaf animals *n* = 6.

Group 3: *GluA2/3* AMPA R subunits immunocytochemistry. Control group *n* = 4; bilateral deaf animals *n* = 4.

Group 4: *GAD67* and *PV* immunocytochemistry. Control group *n* = 3; bilateral deaf animals *n* = 5.

### Surgery

Surgery and hearing loss validation in this animal model of long-term hearing deprivation have been extensively detailed in a previous paper published by our laboratory (Pernia et al. 2017). Deep anaesthesia was induced by intramuscular injections of ketamine chlorhydrate (30 mg/kg BW; Imalgene 1000, Rhone Méreuse, Lyon, France) and 5 mg/kg xylazine chlorhydrate (5 mg/kg BW; Rompun, Bayer, Leverkusen, Germany). Animals were placed on a heating pad to maintaining the temperature at 37 °C, protecting the eyes by applying a drop of ophthalmic gel (Methocel^®^ 2%, Omnivision GmbH, Puchheim, Germany). Using a surgical microscope (Wild M650, Wild Heerbrugg, Switzerland), bilateral lesions were performed by puncturing the cochlea through the ears with a sterile straight needle (gauge 20 g) after middle ear ossicular chain removal. The animals were kept on the heating pad maintaining the temperature at 37 °C (Thermostatic blanket low noise, model RTC-1, Cibertec, Madrid, Spain) after surgery until they woke up. Buprenorphine was subcutaneously injected in the back of the rats (0.05 mg/kg; Buprex^®^ 0.3 mg RB Pharmaceuticals Limited, Slough Berkshire, UK) for analgesia 1 h after the surgery and then every 8 h for the following 72 h.

### Deafness assessment: auditory brainstem recordings

Auditory brainstem recordings (ABRs) were performed in deaf and control animals to show the effectiveness of cochlear puncture in inducing profound hearing loss.

In all four experimental groups, bilateral ABRs were performed before surgery, after surgery, and immediately before euthanasia. Recordings were performed under anaesthesia (ketamine-xylazine) using a close-field real-time signal processing (Tucker-Davis Technologies [TDT], System RZ-6, Alachua, Fl, USA) for groups 2, 3 and 4. In group 1, the anaesthesia protocol for ABRs was similar to that used for VEPs recordings (see below).

Three subcutaneous needle electrodes were used for the recordings, placed at the vertex (active electrode), with the mastoid ipsilateral to the stimulated ear (reference) and the mastoid contralateral to the stimulated ear (ground electrode). Sound stimuli of a 5-ms (ms) window with 0.1 ms alternating polarity click with a repetition rate of 11 bursts/s were delivered in 10-dB ascending steps from 10 to 90 dB SPL. The stimuli were performed in close field using a magnetic speaker [TDT-MF1 Multi-Field Magnetic Speaker, Tucker-Davis Technologies (TDT), Alachua, FL, USA] connected through a 10-centimetre-long plastic tube (Tygon^®^ formula 2375, Sant-Gobain Performance Plastics, Akron, OH, USA), inserted into the external auditory canal. This approach resulted in a 1.4-milisecond delay for stimulus arrival at the tympanic membrane. Responses were averaged 1000 times. Evoked potentials were amplified and digitised using a Medusa RA16PA preamplifier and RA4LI head stage [Tucker-Davis Technologies (TDT), Alachua, FL, USA]. The final signal was filtered with a 500-Hz high-pass filter and a 3000-Hz low-pass filter.

**Fig. 1:**
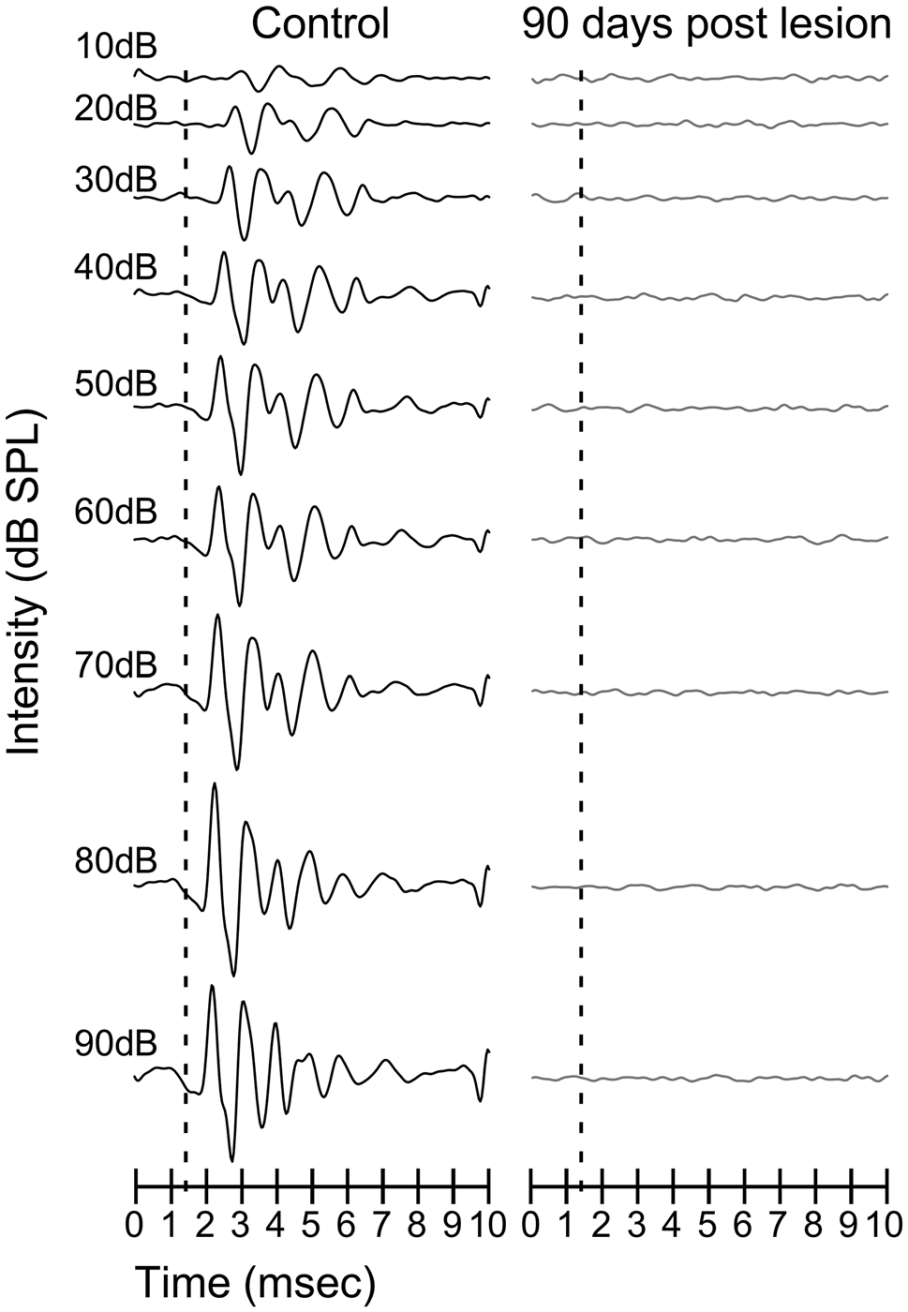
Example of auditory brainstem recordings made before (left) and 90 days after cochlear puncture (90 days post-lesion—right). No waves were detected after click stimulus presentations from 10 to 90 dB, which ensures long-term deafness of the animals in our model of bilateral hearing deprivation

No sound evoked auditory brainstem activity was shown in any of the four deaf animal groups (for an example, please see Fig. 1).

### Visual evoked potentials

Animals from group 1 were placed in a cage to induce gas anaesthesia by applying 5% sevoflurane. Once the hind paw and corneal reflexes disappeared, the animals were placed in a stereotaxic frame to implant stainless screws (1.2-mm diameter, 4-mm length, Stoelting Co., Wood Dale, IL, USA). A small guide hole was created using a hand drill; screwing was then started by lowering the screws through the bone without touching the cortex and permanently attaching them with dental acrylic cement (DuraLay, Reliance, Dental Mfg. Co., Worth, IL, USA). Pedestal provided an excellent and stable signal-to-noise ratio and avoided potential movement during the experiments (Makowiecki et al. 2015).

The active electrode was placed on the monocular VC coordinates (AP: − 7.5; ML 3.5), and the reference electrode was placed in the contralateral hemisphere (AP: − 5.5; ML 3.5).

EEG recordings started a week after screw implantation and 90 days after cochlear puncture. EEG recording in the control group were made after the same time lapse as that of the bilateral deaf group. Animals were anaesthetised (1.5–2% of sevoflurane) to maintain stable slow-wave activity. The body temperature was maintained at 37 °C using a homeothermic blanket system with a rectal thermometer probe (Harvard Apparatus, Holliston, MA, USA). For VEP recordings, the animals were placed in a dark cage and allowed to adapt to the darkness for 5 min. Visual stimulation consisted of flashes at 0.5 Hz. VEPs were obtained after averaging 150 stimuli. A differential amplifier (Model 1700 A-M System, LLC, Carlsborg, WA, USA) was used to continuously record the EEG signal. The signal was amplified (× 1000), filtered (1–500 Hz), digitized at 20 kHz by a 1401 CED A/D convertor card (Cambridge Electronic Design, UK) and stored using Spike 2 software (Cambridge Electronic Design, UK) in a PC for online checking and subsequent analysis. N1 wave latency was defined as the time in seconds (s) from the stimulus onset to the positive peak of the wave. The amplitude of wave N1 was measured as the peak-to-peak amplitude between the preceding negative to the subsequent N1-positive peak.

### Tissue extraction for RT-qPCR gene expression analysis

**Fig. 2:**
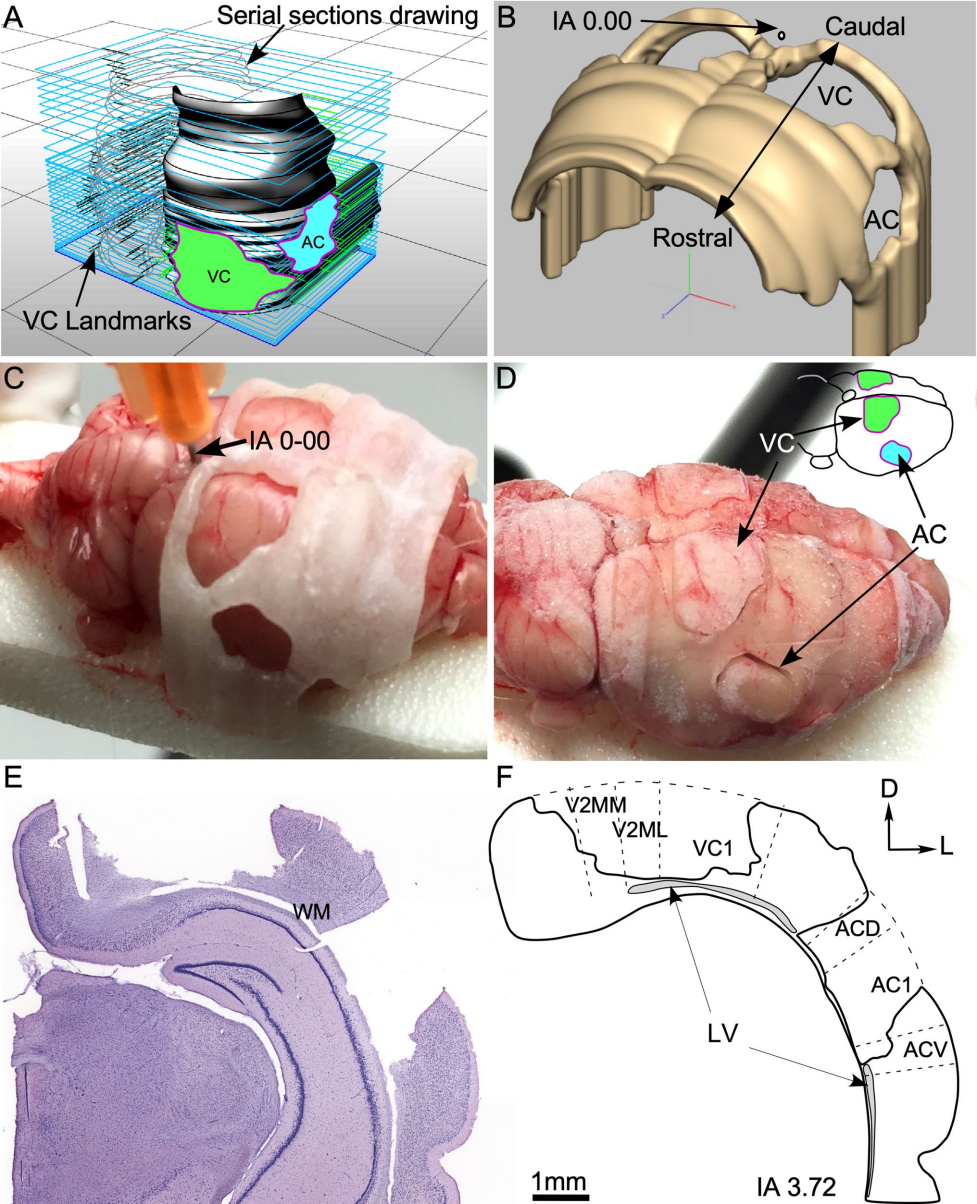
Method for printing a 3D mold of the rat brain, designed for precise and controlled tissue extraction of separate samples from the auditory (AC) and visual (VC) cortices. **a** Digitised stacked drawings from coronal serial sections obtained from Paxinos atlas. Please note the holes defined for delimiting AC (in blue) and VC (in green) in the stack reconstruction. **b** Resulting solid model after 3D printing of the mask. Surface holes precisely delimit AC and VC. **c** Tissue extraction procedure. Printed mask was placed carefully on the brain surface. For defining the correct position of the matrix in relation to stereotaxic coordinates, a guide needle was inserted in interaural (IA) 0.00 in the brain (arrow). **d** Mask and brain together were immersed in liquid nitrogen. After careful demarcation with a small scalpel, AC and VC (arrows) were delineated on the surface of the brain before tissue extraction. **e** Method for checking the accuracy of tissue extraction. Example of a coronal Nissl-stained section after AC and VC extraction guided by the 3D mask. **f** Superimposition to the section silhouette of cytoarchitectural landmarks taken from an equivalent IA section of Paxinos atlas. This approach makes it possible to confirm the precision and accuracy of the extraction of the two sensory cortices in separate samples for RT-qPCR analysis. *AC1* primary auditory cortex, *ACD* dorsal auditory cortex, *ACV* ventral auditory cortex, *VC1* primary visual cortex, *V2MM* secondary medio medial visual cortex, *V2ML* secondary mediolateral visual cortex, *LV* lateral ventricle, *WM* white matter

Animals from group 2 were anaesthetised and decapitated and, after quick brain extraction (less than 4 min), the AC and VC were delimited by superimposing a 3D printed mask. The mask was designed using digitalised drawing lines of coronal serial sections from the Paxinos and Watson atlas (from IA 0.12 to 12.24) (Fig. 2a, b). A plastic brain matrix was digitally built after delimiting AC and VC landmarks in stereotaxic atlas digital pictures (Design X^®^ program) and printed with a 3D System 3510SD (Visijet M3 Crystal). The 3D plastic matrix was positioned carefully on the brain surface using a guide needle inserted in the brain in IA 0.00 (Fig. 2c). After being quickly frozen in liquid nitrogen, both brain and plastic matrix, AC and VC from both hemispheres were precisely extracted using a small (0.7 mm) ad hoc trowel (Fig. 2d). Subsequently, AC and VC tissues were stored at − 80 °C until use.

To ensure the accuracy of AC and VC tissue extraction, coronal serial sections of brains after tissue extraction were stained with 1% cresyl violet for 10 min (C-violet 1791; Sigma-Aldrich Co., St. Louis, MO, USA). Staining differentiation was in 96% alcohol + acetic acid and sections were finally dehydrated in increasing concentrations of alcohol from 50% to 100%, followed by clearing in xylene (3 min). Stained sections were photographed using a Leica DMRB microscope and the “Virtual slice” module of Neurolucida 8.0 (MBF-Bioscience, Williston, Vermont, USA) (Fig. 2e). Photomicrographs were superimposed with landmarks from Paxinos and Watson atlas coordinates (2005) to determine dorso-ventral and rostro-caudal limits of the extracted AC and VC (Fig. 2f). All areas of extraction (holes with loss of tissue in the Nissl stained sections) were shown inside the coordinates of visual and auditory cytoarchitectural limits (Fig. 2e, f).

### RNA isolation

Total RNA was purified from the collected and homogenised cortices to study the gene expression of target mRNAs using RNAqueous-micro kit^®^ (Invitrogen, Thermofisher, Carlsbad, CA, USA), performed according to the manufacturer’s instructions. RNA concentrations and quality were assessed using an RNA 6000 Nano LabChip kit (Agilent Technologies, Palo Alto, CA, USA) and an Agilent 2100 Bioanalyzer to assess the integrity of the 18S and 28S rRNA bands, as well as an RNA integrity number (RIN), with 0 corresponding to fully degraded RNA and 10 corresponding to intact RNA. For all RT-qPCR analyses, only RNA samples with a RIN of at least 7.5 were used; most samples had a RIN of at least 8.0, thus fulfilling one of the requirements of an optimal RT-qPCR experiment according to Fleige et al. (2006).

### Quantitative reverse-transcription real-time PCR (RT-qPCR)

Total RNA (1 µg) primed with oligo-dT and random hexamer primers was reverse-transcribed into cDNA at 37 °C for 2 h using the first-strand cDNA synthesis kit (Promega Corporation, Madison, WI, USA) in a 20 µl volume and stored at − 20 °C until use, according to the manufacturer’s instructions. In all cases, a reverse transcriptase negative control was used to test for genomic DNA contamination.

RT-qPCR was performed using the SYBR-Green method with a 2× master mix (Applied Biosystems, Thermofisher, Carlsbad, CA, USA). Each reaction contained 10 µl of Master Mix, 0.8 µl of each primer, 3 µl of each cDNA sample in a different serial cDNA quantity for each gene, and Milli-Q water up to 20 µl. The amplification reaction was performed in the QuantStudio 7 Flex Real-Time RT-qPCR System (Applied Biosystems, Thermofisher, Carlsbad, CA, USA) with the following conditions: 10 min at 95 °C, followed by 40 cycles of 15 s at 95 °C and 30 s at 60 °C depending on each pair of primers. Three RT-qPCR reactions were performed for each sample per plate, and each experiment was repeated twice. An RNA-free (negative) control sample was also used. The list of primers used is provided in Table 1. All primers had at least one primer crossing an exon–exon boundary and were designed to have similar melting temperatures and to give similar amplicon sizes.

**Table 1:** RT-qPCR primers used in gene expression analysis

Target protein	Number GenBank^a^	Primer Forward	CDNA Forward ^a^	Primer Reverse	CDNA Reverse^a^	Size of products	*E*
*c*-*Fos*	NM_022197.2	ACGGAGAATCCGAAGGGAAA	569–588	TGTCTCCGCTTGGAGCGTAT	640–659	91	91.2
*Arc/Arg3.1*	NM_019361.1	AGGCAGCAGCTGGAGTCTTC	2102–2121	GGGTGGTACCCCTTCCAGAC	2176–2195	94	97.77
*GluA2*	NM_017261	CGGCAGCTCAGCTAAAAACT	173–192	TTGTAGCTGGTGGCTGTTGA	243–262	90	96.30
*Gad67*	NM_017007.1	CGTTTGATCCGATCCAGGAG	1235–1254	GAGTTTGTGGCGATGCTTCC	1331–1350	116	98.66
*Gad65*	NM_012563.1	GATCGGAACAGACAGCGTGA	953–972	GTGGCACTCACCAGGAAAGG	1059–1078	126	95.72
*Gabra1*	NM_183326.2	TCAGCAAAATCGACCGACTG	1468–1487	GTGGGGGCTTTTAGCTGAGG	1560–1579	112	98.47
*Gabr2*	NM_012957.2	GCCTCCAGCATCCAGTATCG	1192–1211	GTCGGGGATGGTGATTTTCA	1307–1326	135	91.30
*Rpl19*	NM_031103	TCGCCAATGCCAACTCTCGTC	123–143	AGCCCGGGAATGGACAGTCAC	191–211	89	96.48

^a^Primary location in the corresponding GenBank sequences of rat origin is indicated

^b^RTqPCR primer efficiency (*E*) was calculated according to the following equation: *E*: 10^(−1/slope)^

To identify the most stable gene as an endogenous reference for RT-qPCR data normalisation, three candidates [β-actin (*Actb*), glyceraldehyde 3-phosphate dehydrogenase (*Gapdh*) and ribosomal protein L19 (*Rpl19*) (Invitrogen, Thermofisher, Carlsbad, CA, USA)] were selected, and their expression was measured by RT-qPCR. The Norm-Finder software (Andersen et al. 2004) was used to calculate intra- and inter-group expression. The mean of the threshold cycle (Ct) value and the primer efficiency value of *Rpl19* were used for normalisation.

The comparative Ct method was used to obtain quantitative data (Schmittgen and Livak 2008). After removing outliers, according to Burns et al. (2005), raw fluorescence data were used to determine the RT-qPCR amplification efficiency (*E*) according to the equation *E* = [10^(−1/slope)^ − 1] × 100. All amplifications had an *E* value of 100 ± 10%; an *E* value close to 100% indicates efficient amplification. The relative gene expression value (fold change, FC) of each transcript was calculated according to equation 2^−(ΔCt “condition 1” − ΔCt “condition 2”)^, where “condition 1” corresponds to experimental samples (90 dpl), “condition 2” to the samples of control animals, and the ΔCt of each “condition” is *C*_t“experimental gene”_ − *C*_t“endogenous gene”_ (Livak and Schmittgen 2001; Schmittgen and Livak 2008). The standard deviation of each relative level of gene expression was calculated as a measure of data variation.

### Immunocytochemistry

Animals of groups 3 and 4 were deeply anesthetised with an intraperitoneal injection of 6% sodium pentobarbitone (60 mg/kg BW) and perfused transcardially with 4% p-formaldehyde in 0.1 M phosphate buffer (PB).

Brains were dissected, post-fixed and serially sectioned in the coronal plane with a sliding freezing microtome (HM 430 Sliding, MICROM International, Waldorf, Germany). Coronal serial sections (40 μm thickness) were immunostained for GluA2/3 AMPA R subunits in group 3, and in alternate serial sections for GAD67 and PV in group 4 (for a description of the antibodies see Table 2).

**Table 2:** Summarised description of antibodies used

Antigen	Immunogen	Description	Dilution used
GluA2/3	Carboxy terminus peptide of rat GluR2 conjugated to BSA with glutaraldehyde (EGYNVYGIESVKI)	Polyclonal Rabbit, Merck Millipore #AB1506; RRID:AB_90710	1:100 TBS 0.05 M + Triton-Tx 0.3%
GAD67	Recombinant GAD67 protein	Monoclonal mouse, Merck Millipore #MAB5406 clone 1G10.2 RRID:AB_2278725	1:1000 TBS 0.05 M + Triton-Tx 0.3%
Parvalbumin	Rat muscle parvalbumin	Polyclonal Rabbit, Swant #PV-25; RRID:AB_10000344	1:5000 TBS 0.05 M + Triton-Tx 0.3%

Group 3: GluA2/3 AMPA R subunits immunocytochemistry. Control group *n*: 4; bilateral deaf animals *n*: 4

Group 4: GAD67 and PV immunocytochemistry. Control group *n*: 3; bilateral deaf animals *n*: 5

Our methodological approach was designed to examine markers investigated in parallel for gene expression by RT-qPCR and for protein expression by quantitative immunocytochemistry.

In our staining procedure, paired sections from control and deaf animal groups were processed simultaneously to limit confusing differences in grey level measurements caused by immunocytochemical processing. Free-floating sections were washed in 0.1 M PB pH 7.6, and endogenous peroxidase activity was subsequently inhibited by incubation in 10% methanol + 3% H_2_O_2_ in 0.1 M PB for 10 min. Sections were washed in PB and 0.05 M Tris-buffered saline, pH 8.0 + Triton X-100, 0.5% (T9284 Sigma, St. Louis, MO, USA; TBS-Tx). Sections were then incubated in primary antisera (Table 2), for 48 h at 4 °C. Nonspecific labelling was blocked using fetal calf serum (10%). After washing three times in TBS-Tx, for 15 min, all sections were incubated with an anti-rabbit biotinylated secondary antibody (biotinylated anti-rabbit IgG H1L, BA-1000; Vector, Burlingame, CA, USA) and with an anti-mouse biotinylated secondary antibody (biotinylated anti-rabbit IgG H1L, BA-2000; Vector, Burlingame, CA, USA) at 1:200 dilution in TBS-Tx for 120 min at room temperature. Sections were then washed with TBS-Tx and incubated for 180 min in avidin/biotin–peroxidase (ABC complex, Vectastain Standard ABC kit PK-4000; Vector, Burlingame, CA, USA) and further washed with TBS-Tx, followed by Tris HCl, pH 8.0. They were then incubated in 3,3-diaminobenzidine tetrahydrochloride (DAB; D-9015; Sigma-Aldrich, St. Louis, MO, USA) with 0.006% H_2_O_2_ and 0.4% nickel ammonium sulphate to visualise the peroxidase reaction. One section per case was used as a negative control, by processing without the primary antibody to test the specificity of the immunostaining detection system.

### Quantitative immunocytochemistry

Panoramic mosaics of the entire cortex were captured from six coronal sections for each animal at interaural coordinates (IA): 0.96, 2.16, 3.00, 4.20, 4.44, and 4.80 corresponding with the Paxinos and Watson cytoarchitectural stereotaxic atlas. Digital photomicrographs (mosaics) were taken using a Leica DMRB microscope with a ×10 objective (Leica Plan Apo). Mosaics were taken and assembled using the “Virtual slice” module of Neurolucida 8.0 (MBF-Bioscience, Williston, Vermont, USA) and adjusting the microscope illumination source before each image capture, using a stepped density filter (11 levels) (^®^Eo Edmund industrial optics—ref 32599, Karlsruhe, Germany).

Photomicrographs of GluA2/3 AMPA R subunits and GAD67 immunostained sections were analysed with Image J 2.0 software (USA; RRID:SCR_003070), using the default thresholding segmentation to measure optical density (OD) separately in primary and secondary ACs and VCs. After measuring the OD of the cytoarchitectural subdivisions as a whole, cortical layers were digitally cut and analysed separately.

As a reference for OD analysis, photomicrographs of CA1, CA2, CA3 and the dentate gyrus of the hippocampus were made from 3 coronal sections for each animal at 5.40 IA. The normalised hippocampal control OD for GluA2/3 and GAD67 immunoreactivity showed no significant difference between control and deaf animals (Figs. 6a, 8a).

PV immunostained sections displayed well-defined contours of neurons (good background-to-noise ratio), which enabled a clear delineation of individual neurons by density gradient segmentation. Segmentation for PV immunoreactive sections was performed using the maximum entropy threshold algorithm (Gull and Skilling 1984; Bardera et al. 2009). The number of segmented neurons was normalised to N/10,000 µm^2^.

The mean and standard deviation of grey levels was assessed globally in all sections to cancel out differences in immunostaining intensities within and among cases. Cases with average grey levels above or below the total mean grey level ± the standard deviation (SD) were eliminated from the analysis (only in one case from group 3).

The boundaries of AC and VC were defined according to Paxinos and Watson atlas coordinates (Paxinos and Watson 2005). We followed Palomero-Gallagher and Zilles guidelines (Paxinos 2004) to define the AC and VC layers at the rostro-caudal level. Quantitative values of each section from every case were averaged separately for each sensory cortex: primary AC (AC1), secondary dorsal AC (ACD), secondary ventral AC (ACV), primary VC (VC1), secondary lateral VC (V2L) and secondary medial VC (V2M, which contain V2MM and V2ML).

### Statistical analysis

Statistical analysis was performed using the IBM^®^ SPSS^®^ software, version 25.0.0.0 (IBM Corp. and SPSS Inc., Chicago, IL, USA, RRID: SCR_002865).

To identify significant differences in VEPs wave amplitude (mV) and latencies (s), a student’s *t* test was employed, which is the most common statistical test used to analyse to independent samples. The results were considered significant at *p* < 0.05. The significance of the RT-qPCR analysis results was determined using a two-tailed *t* test for each gene, considering |FC| > 1 significant when *p* < 0.05.

Differences between groups in the immunocytochemistry were assessed by general linear model univariate analysis followed by the post hoc Scheffe-test and the Bonferroni test. Differences were considered significant at *p* < 0.05.

## Results

### Electrical evoked activity

By showing flat waves in ABRs with 90 dB click stimulation, we ensured sensory deprivation at 90 days after cochlear puncture (90 days post-lesion, dpl) (Fig. 1).

**Fig. 3:**
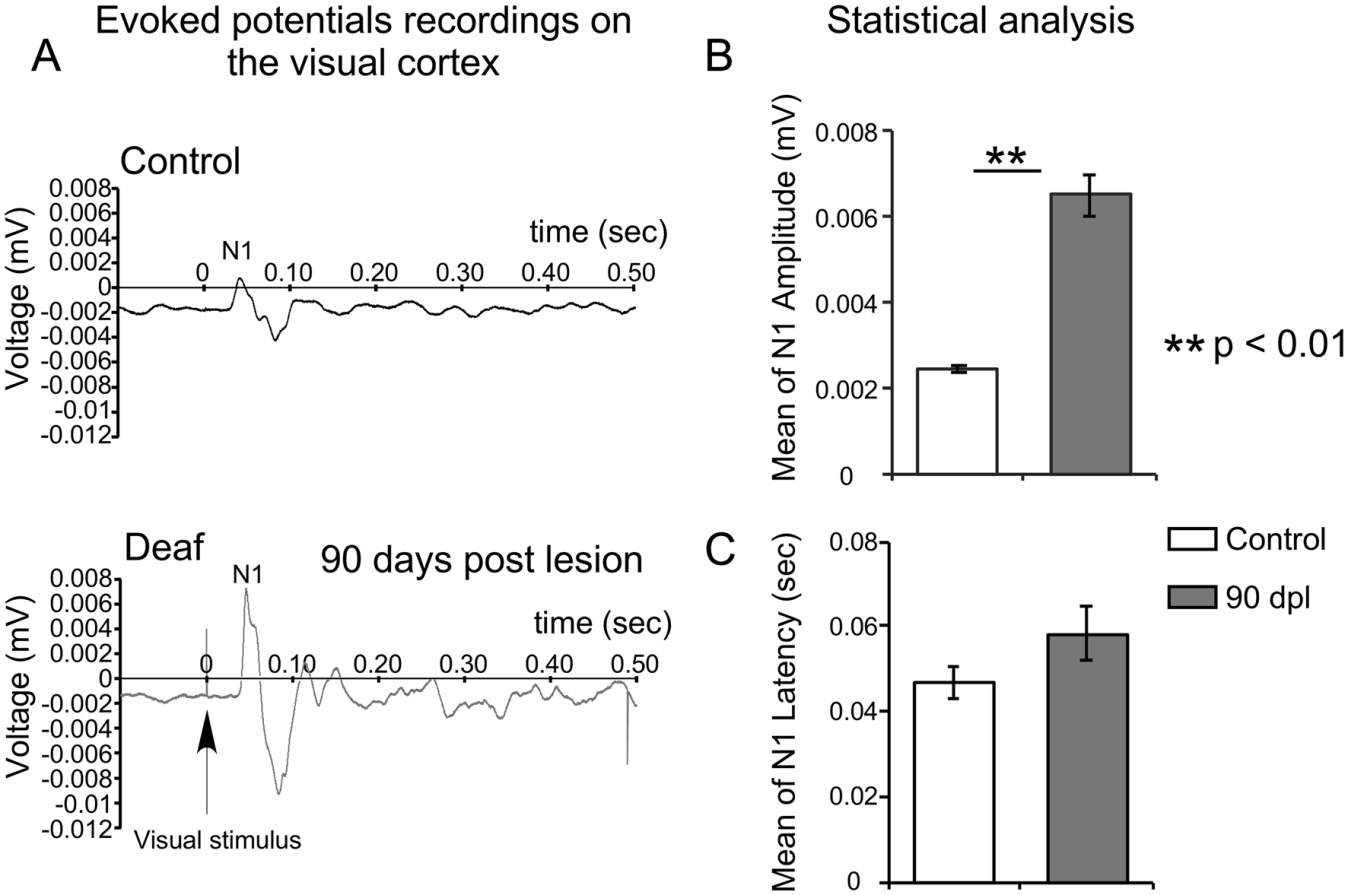
**a** Representative VEP recording of control (top) and deaf (bottom) animals at 90 days post-lesion (90 dpl). N1 wave amplitude (by peak to peak voltage measurements) increases three-fold after long-term deafness. **b** Graphic representation of the mean N1 wave amplitude, standard error and statistical analysis of N1 after hearing deprivation (control animals *n* = 3; deaf animals *n* = 6; **p < 0.01). These data confirm a significant increase in N1 wave amplitude in our animal model. **c** Bar representation of mean N1 wave latencies, standard error and statistical analysis of N1 after hearing deprivation (control animals *n* = 3; deaf animals *n* = 6; **p < 0.01)

VEPs from VC after a repetitive flash stimulation protocol allowed us to distinguish primary (P1-N1) from secondary (P2-N2-P3) components (Fig. 3a), as described by Creel et al. (1974). Figure 3a shows representative VEPs from a control animal (top) and from a deaf animal at 90 dpl (bottom); the increase in VEP in the deaf animal is evident by visual comparison.

For the whole population (*n* = 6), a significant increase of 156.39% was shown after comparing mean amplitudes between control and bilateral deaf animal groups (deaf animals mean N1 wave amplitude ± SE = 0.0062 ± 0.0004 mV versus 0.0024 ± 0.000022 mV in control ones) (Fig. 3b). No differences in N1 mean latency were found between groups (Fig. 3c). These results are compatible with a long-term increase in VC activation in deaf animals.

### Gene expression in the auditory and visual cortices

Tissue samples were collected from the AC and VC using our 3D printed mask (please see “Materials and methods”) (Fig. 2). After superimposing drawings from the Paxinos atlas over the Nissl staining sections, empty areas of tissue extraction were always shown inside the cytoarchitectural limits of AC and VC (Fig. 2e, f). This approach allowed us to collect tissue samples for RT-qPCR analysis of the expression levels of a selected group of genes (*Arc/Arg3.*1, *c*-*Fos*, *GluA2*, *Gad65*, *Gad67, Gabra1*, *Gabbr2* and *Parvalbumin*) separately for the AC and the VC.

**Fig. 4:**
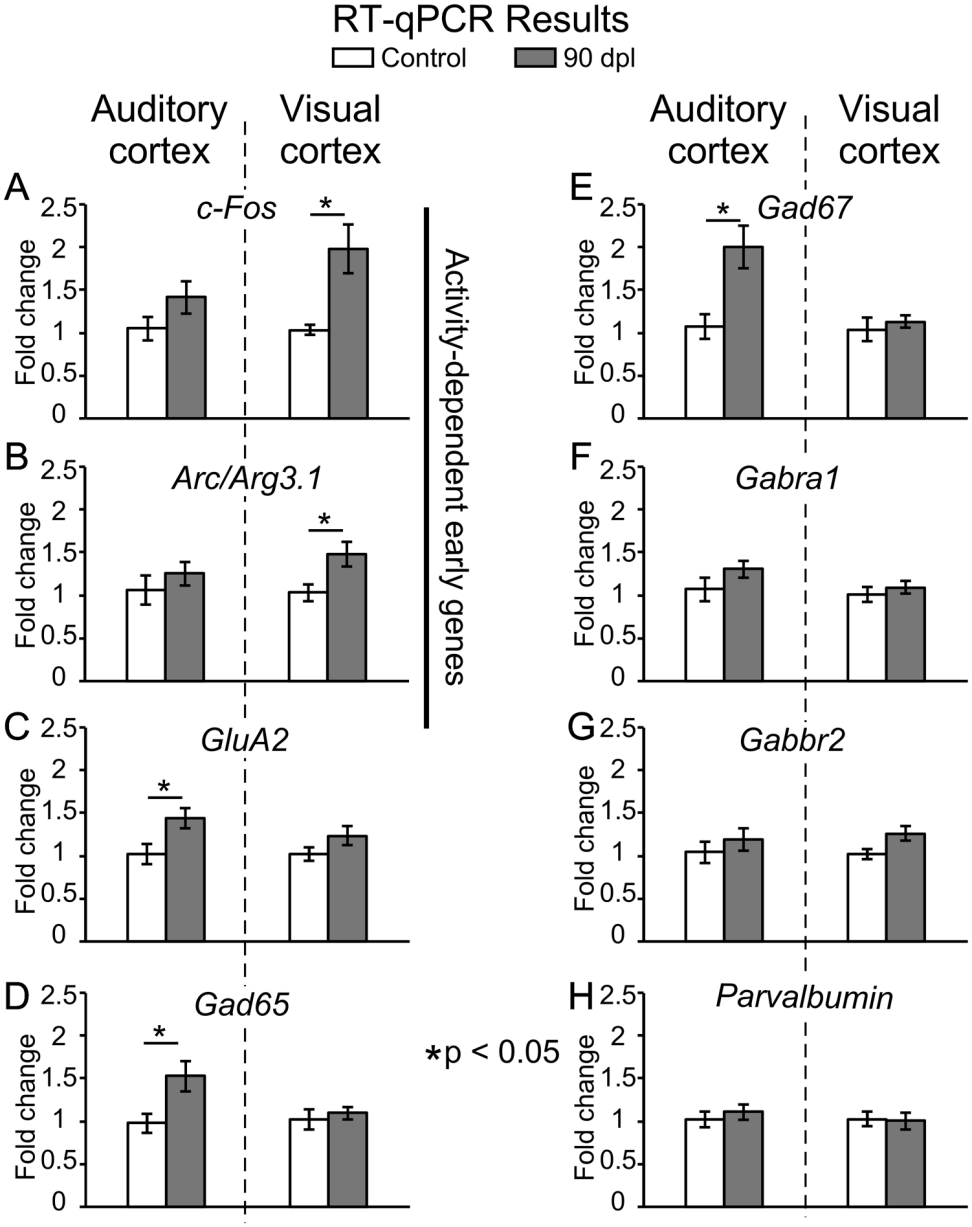
Bar charts illustrating changes in mRNA levels of genes in control (white columns) and deaf group (grey columns) at 90 days post-lesion (90 dpl). Results are expressed as mean ± standard error of fold change. Asterisks represent significant differences between groups (*p < 0.05). Activity-dependent early genes **a***c*-*Fos* (*p* = 0.021) and **b***Arc/Arg3.1* (*p* = 0.04). **c***GluA2* AMPA receptor (AMPA R) subunit (*p* = 0.035), **d***Gad65* (*p* = 0.03), **e***Gad67* (*p* = 0.017), **f***Gabra1* subunit, **g***Gabb*-*r2* subunit, **h***Parvalbumin*

In the deaf animals of experimental group 2, the expression level showed a significant increase in *c*-*Fos* (fold change 1.97; *p* = 0.021) (Fig. 4a) and *Arc/Arg3.1* (fold change 1.47; *p* = 0.04) in the VC (Fig. 4b). The expression levels of the *GluA2* AMPA R subunit showed a significant increase of 1.43-fold (*p* = 0.035) in the AC but not in the VC (Fig. 4c). *Gad65* and *Gad67* levels significantly increased 1.53- (*p* = 0.03) and 1.99-fold (*p* = 0.017) in the AC of the deaf animals from group 2 (Figs. 4d and e), respectively. However, neither the AC nor the VC showed significant changes after RT-qPCR analysis for *Gabra1*, *Gabbr2* or *Parvalbumin* when comparing controls to hearing-deprived animals (Fig. 4f–h).

### Protein expression in the auditory and visual cortices

#### GluA2/3 AMPA receptor subunits

**Fig. 5:**
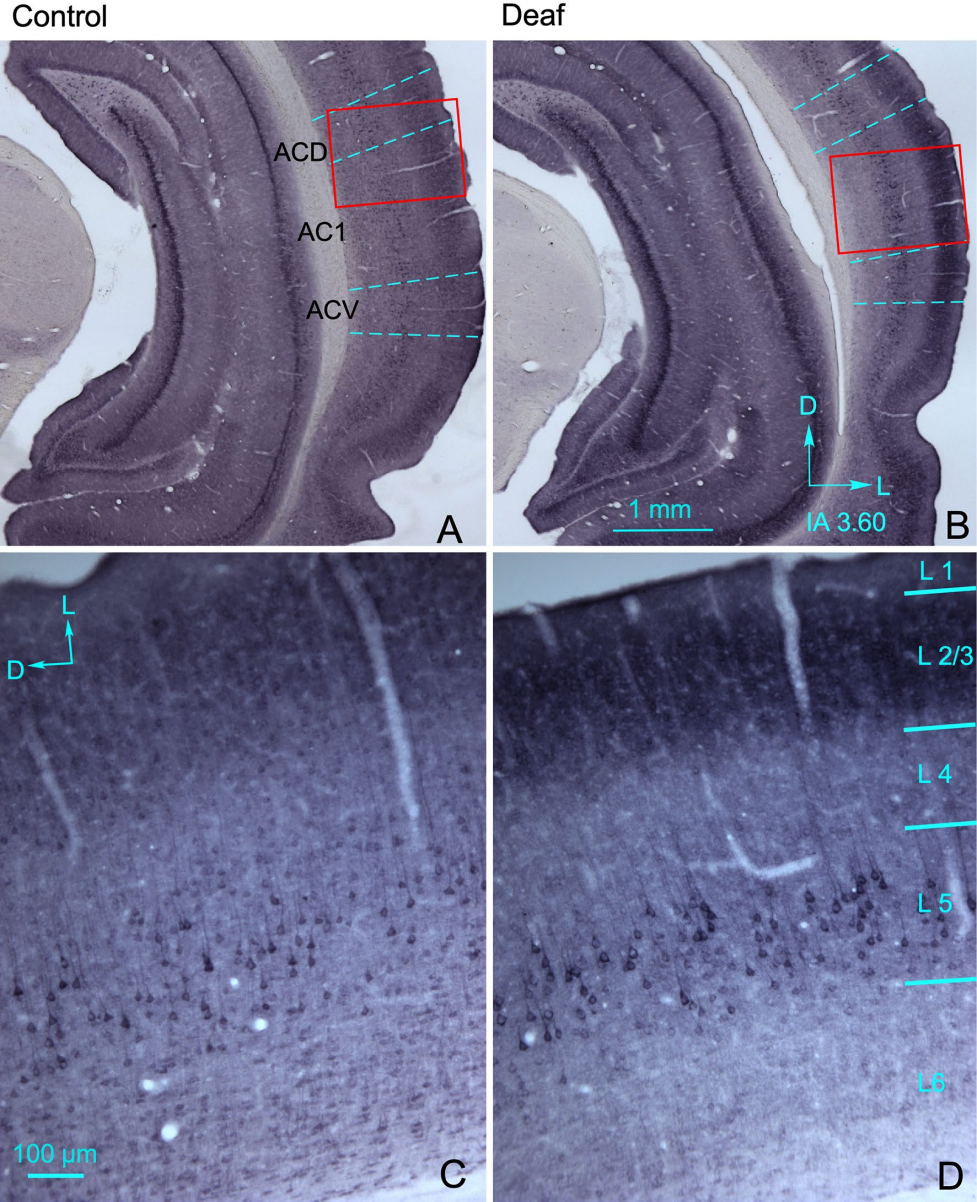
Representative GluA2/3 AMPA receptor (R) subunit-immunostained coronal sections from control (**a**, **c**) and deaf (**b**, **d**) animals at 90 days post-lesion (90 dpl) at an equivalent rostro caudal level (IA 3.60). Dotted blue lines represent cytoarchitectural subdivisions of the auditory cortex (AC). Red rectangles insets, label the AC areas shown at a higher magnification in **c**, **d**. **c**, **d** Details of the primary auditory cortex (AC1). Notice the overall increase in immunoreactivity in layers 2/3 after comparing sections of deprived animals (**b**, **d**) to those of their controls (**a**, **c**). ACD secondary dorsal AC, ACV secondary ventral AC

GluA2/3 AMPA R subunits were observed throughout the cortex with differences in immunostaining which allowed us to easily distinguish layers and cytoarchitectural subdivisions (Fig. 5a, b, dotted lines in coronal sections from control and deaf animal). Immunoreactivity was denser in the soma and dendrites of pyramidal neurons in layers 2/3 when comparing the AC1 of deaf animals (Fig. 5d) to that of controls (Fig. 5c).

**Fig. 6:**
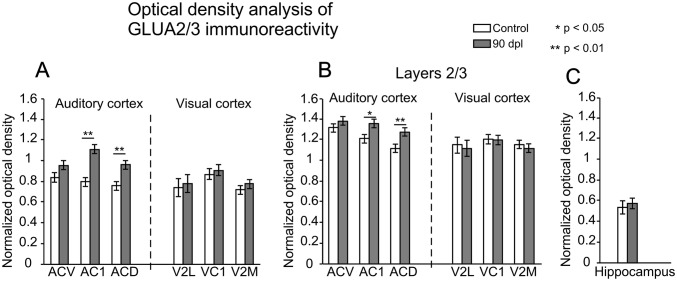
**a** Changes in normalised optical density (OD) of GluA2/3 AMPA R subunits immunoreactivity in auditory (AC) (left) and visual (VC) (right) cortices of control (white columns) and deaf (grey columns) animals. Quantitative analyses were performed separately for primary AC1, secondary ventral AC (ACV), secondary dorsal AC (ACD), primary (VC1), secondary lateral VC (V2L) and secondary medial (V2M, which contain V2MM and V2ML) VC. Each subdivision analysed as a whole. **b** After separately analysing by laminae layers 2/3 show significant differences in AC1 and ACD. Asterisks indicate significant differences between groups (*p < 0.05; **p < 0.01). Results are expressed as mean ± standard error. Sections from each experimental group were incubated simultaneously with their own controls to cancel out differences in intensity measurements caused by differences in processing. **c** Bar chart illustrate non-significant differences for normalised OD values of GluA2/3 AMPA R in the hippocampus when comparing controls to deaf animals

Among all cortical primary and secondary subdivisions analysed, only the OD values of AC1 and ACD were significantly increased in deaf animals (Fig. 6a). However, no significant differences were found in any of the other cortices analysed when comparing controls to deaf animals (Fig. 6a). The OD values of layers 2/3 in AC1 and ACD subdivisions were significantly higher in deaf animals than in controls (Fig. 6b). No significant differences were found in the other layers. Densitometry control, by hippocampal OD analysis, showed no significant differences (Fig. 6c).

### Densitometry control, by hippocampal OD analysis, showed no significant difference (Fig. 6c)

#### GAD67

**Fig. 7:**
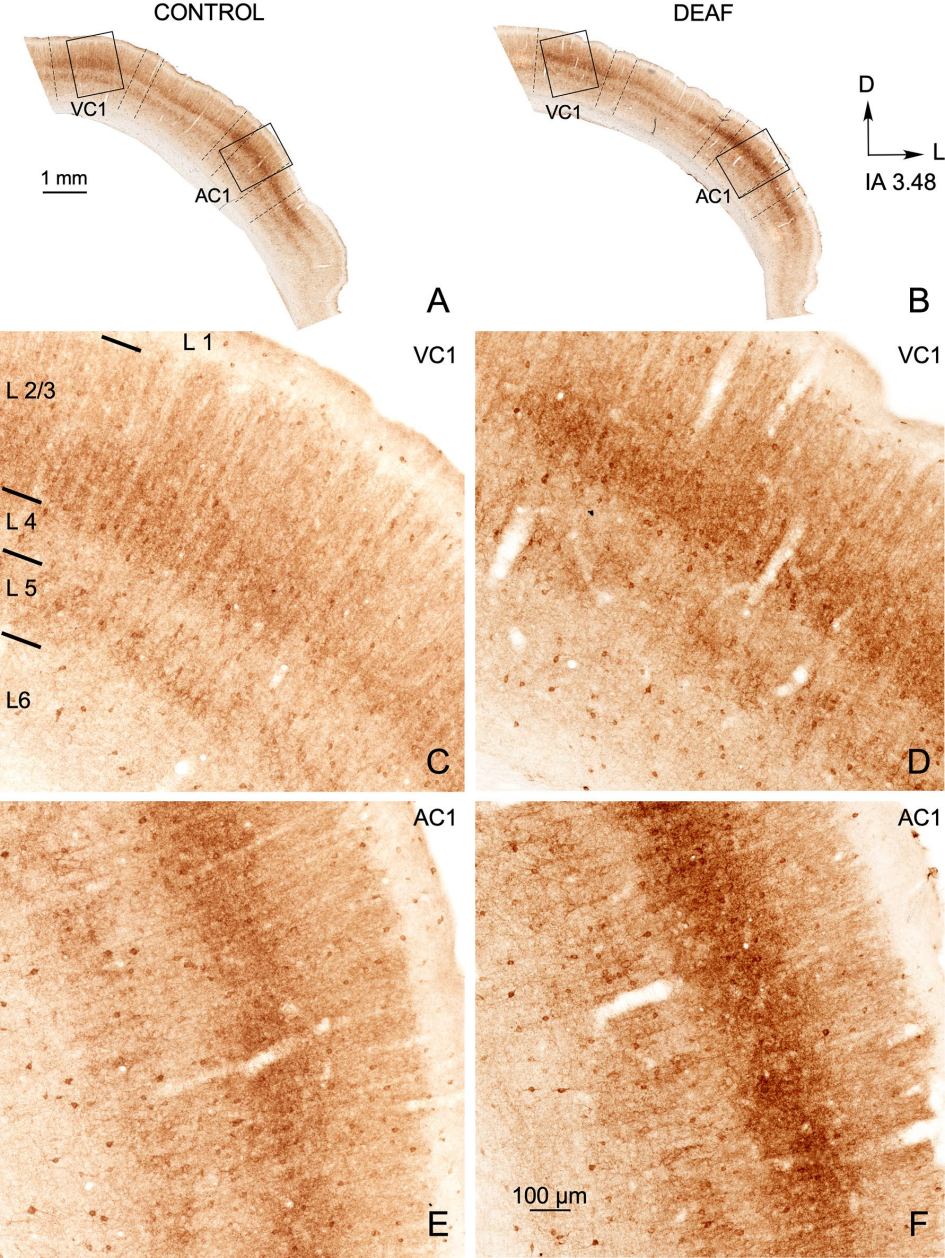
Panoramic view of GAD67 immunostained coronal sections from control (**a**, **c**, **e**) and deaf (**b**, **d**, **f**) animals at an equivalent rostro-caudal level (IA 3.48). Denser immunoreactivity in layers 2/3, facilitates the delimitation of cytoarchitectural cortical subdivisions (borders defined by dotted lines) and layers. Rectangles in **a**, **b,** respectively, delimit details shown at a higher magnification in the bottom of the panel. Denser immunostaining in layer 2/3 is noticeable when comparing the primary visual (VC1) and auditory (AC1) cortices of deaf animals (**d**, **f**) to controls (**c**, **e**)

Microscopically, the denser immunoreactive neuropil in layers 2/3 and 5 allowed us to define cortical layers and cytoarchitectural subdivisions along the cortex (Fig. 7a, b). In particular, AC1 and VC1 were easily distinguished because their immunostaining was darker than that of surrounding secondary subdivisions (Fig. 7a, b, in both coronal sections from control and deaf animal).

In sections from deaf animals, qualitatively, AC1 and VC1 exhibited denser immunoreactive neuronal soma and neuropil, mainly in layers 2/3 and 5 (compare Fig. 7c, e with deaf animals in Fig. 7d, f).

**Fig. 8:**
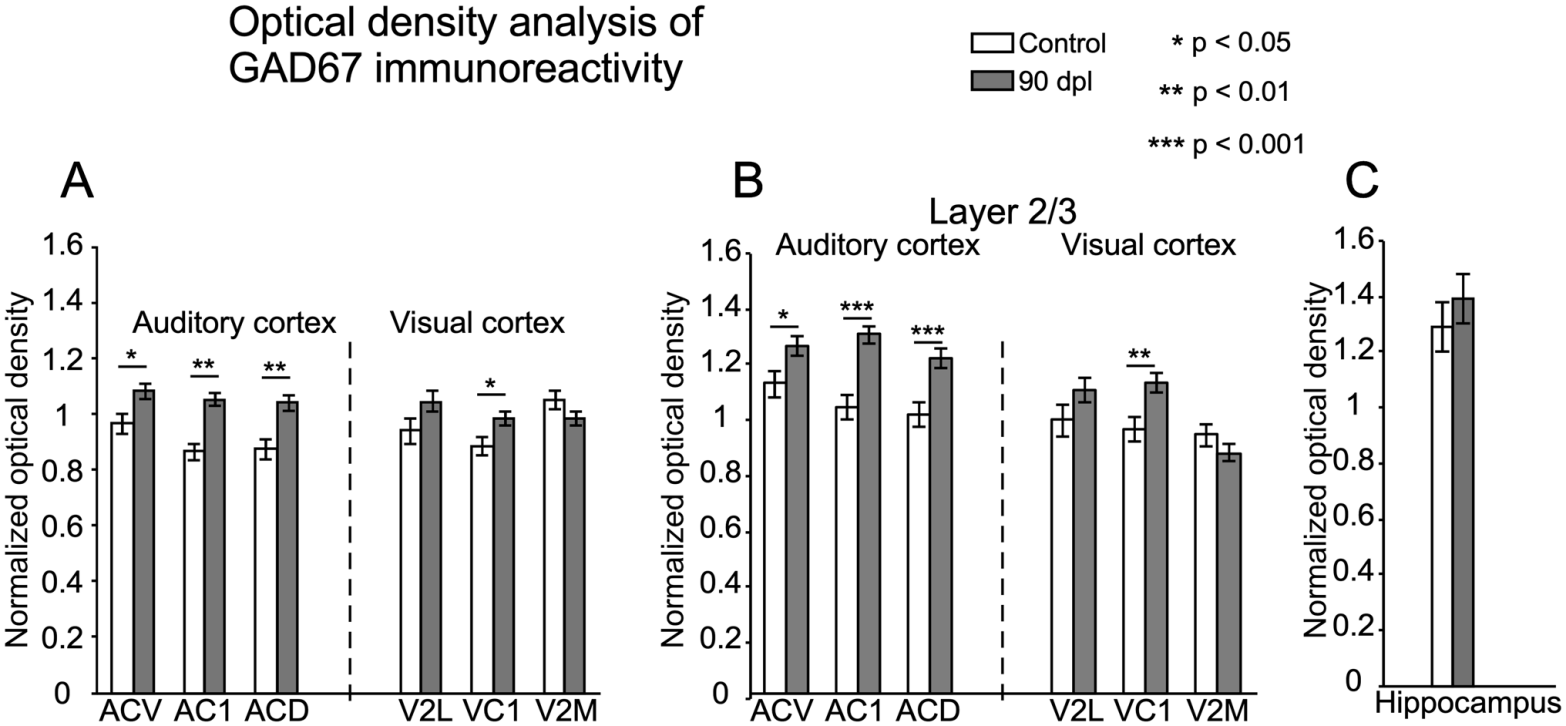
**a** Changes in normalised optical density (OD) of GAD67 immunoreactivity in AC (left) and VC (right) of control (white columns) and deaf group at 90 dpl (grey columns). Quantitative analyses were performed separately for AC1, secondary ventral AC (ACV), secondary dorsal AC (ACD), primary VC (VC1), secondary lateral VC (V2L) and secondary medial VC (V2M, which contain V2MM and V2ML). Each subdivision was analysed as a whole. **b** After separately analysing by laminae, layers 2/3 show significant differences in all the subdivisions of AC and in VC1. Asterisks indicate significant differences between groups (*p < 0.05; **p < 0.01, ***p < 0.001). Results are expressed as mean ± standard error. **c** Bar chart illustrate non-significant differences in normalised OD values of GAD67 in the hippocampus

Quantitative immunocytochemical analysis confirmed that both the primary and secondary areas of AC showed significantly higher values of normalised OD in the deaf animal group (Fig. 8a). However, in the VC, only VC1 showed a significant increase (Fig. 8a).

OD values of layers 2/3 in all subdivisions of the AC (AC1, ACV and ACD) and only in the VC1 were significantly higher in deaf animals (Fig. 8b). No significant differences were shown in any of the other layers. Reference densitometric analysis (hippocampus) did not show any significant differences between groups (Fig. 8c).

#### Parvalbumin

**Fig. 9:**
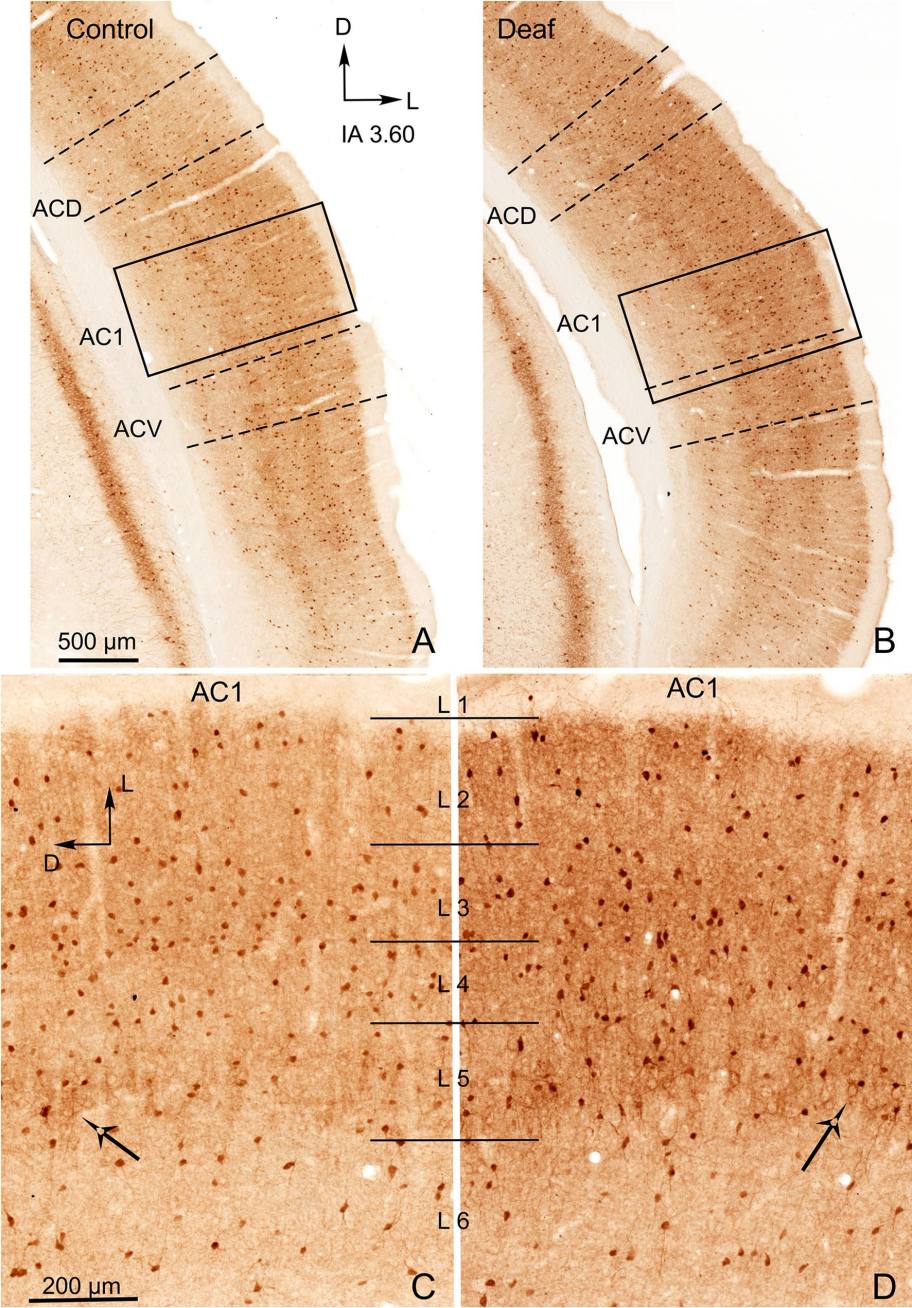
Representative coronal sections immunostained for parvalbumin (PV) from control (**a**, **c**) and deaf (**b**, **d**) animals at an equivalent rostro-caudal level (IA 3.60). Dotted lines in **a**, **b** represent cytoarchitectural subdivisions of the AC. Rectangles in coronal sections in **a**, **b** correspond to details of the primary auditory cortex (AC1) at a higher magnification in **c**, **d**. Notice the higher number of immunoreactive neurons in the microphotograph from the deaf animal in **d**. ACD secondary dorsal AC, ACV secondary ventral AC

**Fig. 10:**
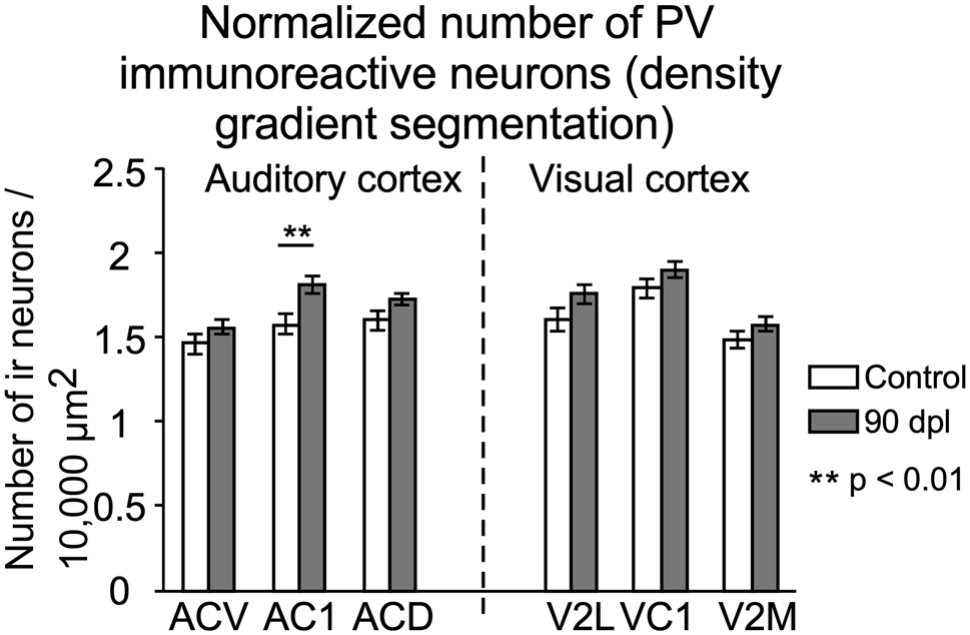
Bar charts illustrate the number of PV-immunoreactive neurons segmented by density gradients in AC (left) and VC (right) of control (white columns) and deaf group at 90 dpl (grey columns). Quantitative analyses were performed separately for AC1, secondary ventral AC (ACV), secondary dorsal AC (ACD), primary VC (VC1), secondary lateral VC (V2L) and secondary medial VC (V2M, which contain V2MM and V2ML). Asterisks indicate significant differences between groups (**p < 0.01). Results are expressed mean ± standard error

Microscopically, PV-immunostained sections showed labelled cell bodies and dendrites, allowing us to identify most stained neurons as medium and large interneurons along layers (Fig. 9a–d). Immunoreactive terminals are usually denser and more abundant in the neuropil of layers 2/3 and 5 (Fig. 9c, d, both in control and deaf animals) and around infragranular pyramidal neurons (Fig. 9c, d, arrows). Immunoreactive somata were observed, homogenously distributed along with cortical subdivisions interspersed across all layers (Fig. 9c, d in AC1 details). In particular, AC1 and VC1 stood out from other cortical subdivisions, including secondary cortices, by their denser PV immunoreactivity (higher in the AC1). An increase in PV immunoreactivity was observed in the neuropil of AC in the sections from deaf animals (comparison between Fig. 9c, d). Nevertheless, these differences were not significant when morphometrically comparing OD values of the AC and VC subdivisions as a whole. However, the good signal-to-noise (background-to-immunostaining) ratio of PV positive neurons (Figs. 9c and d, AC1 details) facilitated the segmentation of neurons by density gradients. After neuronal counting analysis, a significant increase in PV immunoreactive neurons was shown in the AC1 of deaf animals (90 dpl) (Fig. 10).

## Discussion

**Fig. 11:**
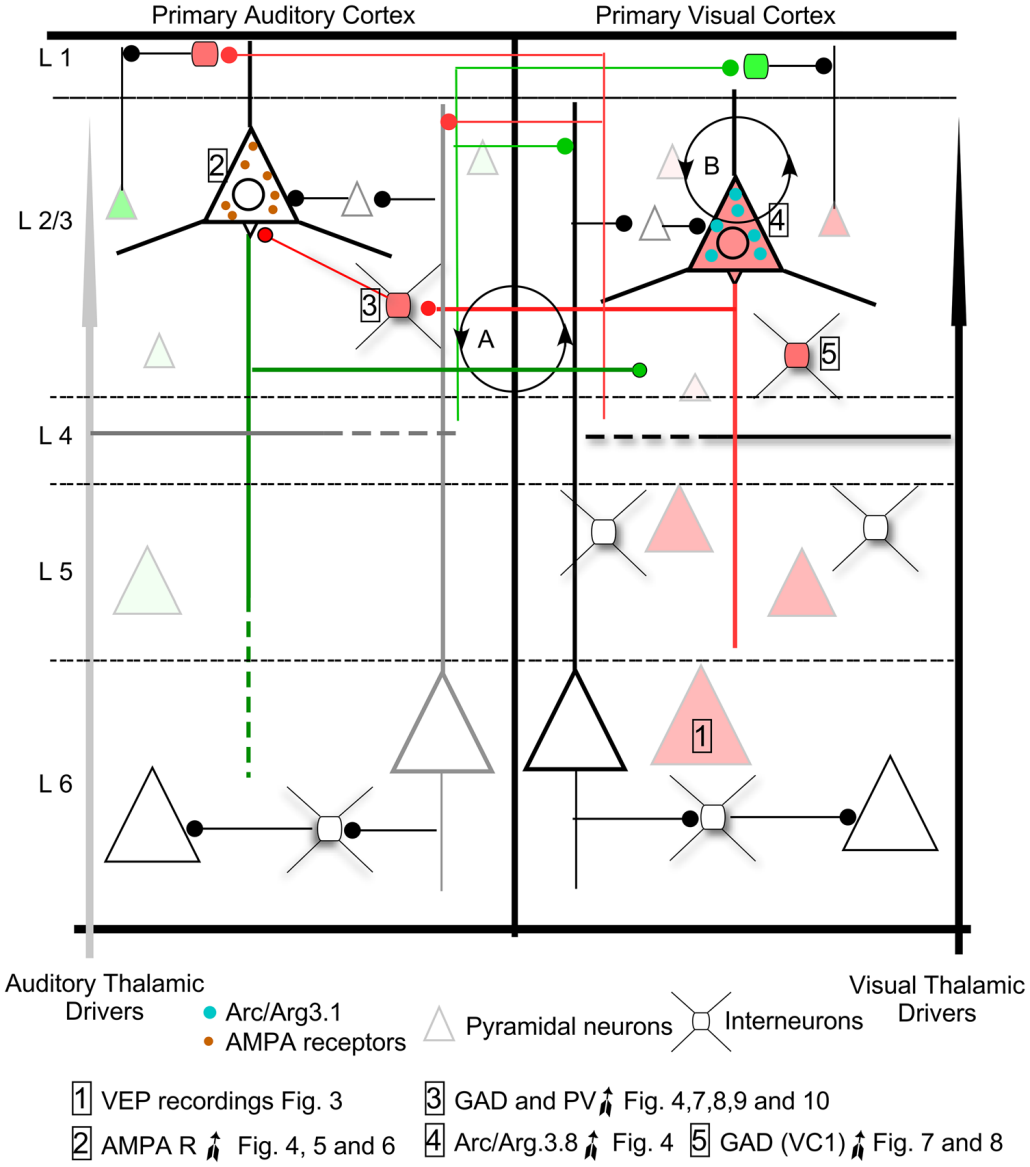
Working hypothesis about cross-modal reaction between auditory and visual cortex after long-term hearing loss. After deafness, altered auditory thalamic inputs induce VC overactivation (1) and increased AMPA R subunit immunoreactivity in layers 2/3 (2). The increase in the number of PV-GABA interneurons in AC suggests increased inhibition of cortical column microcircuits (3). VC over-activation (1) induces a homeostatic compensatory reaction, as shown by increases in Arc/Arg3.1 gene expression (4). In our model, two mechanisms of compensation (labelled in the diagram as circles with arrows A and B) dynamically work in combination: **a** imbalanced reciprocal horizontal connections and **b** increased Arc/Arg3.1 expression in VC, which tend to rebalance VC by internalization of membrane glutamate receptors in pyramidal neurons. Triangles indicate pyramidal neurons and small squares indicate interneurons. Red: more activation and Green: less activation

We demonstrated VC overactivation at 90 days after bilateral cochlear puncture, as shown by significant increases in N1 wave amplitudes of VEP recordings and in c-Fos gene expression (Fig. 11-1. Light red triangles). These functional and structural data suggest imbalanced intermodal processing after deafness. Such plastic reaction is likely triggered by changes in AC horizontal intracortical connections, as shown by significant increases in AMPA receptor and GAD immunoreactivity in layers 2/3 (Fig. 11-2, 3). In addition, a global rebound in AC inhibition was shown by increases in GAD 65 and 67 gene expression and in the number of PV-GABA interneurons (Fig. 11-3. red squares). Secondary intermodal reaction is shown in the VC, with a significant increase in Arc/Arg3.1 and with no significant changes in AMPA R subunits (Fig. 11-4). Analysis of inhibition markers in the VC showed increased GAD immunoreactivity, specifically in the primary VC (Fig. 11-5).

### Auditory cortex plastic reaction

Short-term loss of auditory inputs due to noise overexposure increases the amplitude of auditory evoked potentials in guinea pigs, which suggests functional gain enhancement of the AC after hearing deprivation (Popelár et al. 1987). Similar increases have also been observed in chinchillas after damaging inner hair cells by ototoxicity (Qiu et al. 2000). Hearing loss in cochlear ablated gerbils also raises excitability in layers 2/3 of AC, thus demonstrating increased EPSC amplitude by whole-cell recordings in brain slices (Kotak et al. 2005). Moreover, after visual deprivation in mice, GluA2 and GluA3 gene expression is also up-regulated in the VC (Tropea et al. 2006). Changes in GluA1-GluA2 protein expression, along with increases in the amplitude of spontaneous EPSCs in VC layers 2/3 pyramidal cells, have also been shown after visual deprivation in rats, which indicates increased cortical excitability induced by horizontal intermodal connections (Goel et al. 2006). In the AC of our deaf animals, the cellular stock of GluA2/3 AMPA R subunits is increased in pyramidal neurons of layers 2/3, as shown by the significant higher OD values of immunoreactive products and by microscopic inspection of sections (Figs. 6a, c, 11-2). This increase in GluA2/3 immunoreactivity in AC layers 2/3 could be a homeostatic response resulting after the loss of thalamic drivers induced by deafness in combination with an imbalanced intermodal interaction from the over-activated VC.

In this study, we collected well restricted samples from the VC and AC using a 3D printed mask superimposed on the brain surface. After applying this method, and testing the extraction in serially Nissl sections, we were able to analyse separately both cortices. Consequently, the results of significant fold changes in GAD reflect an extensive increase in GABA gene expression and potentially in GABA synthesis, because all neurons from primary and secondary cortices are present in our samples. *Gad65* and *Gad67* gene expression up-regulation exclusively in the AC (Fig. 4d, e) and increased immunoreactivity of GAD 67 and PV (GABA) interneurons (Fig. 11-3) indicate a rebound in inhibition in the AC. Layers 2/3, specifically denser in GAD67 immunocytochemistry, are the main recipient for horizontal cross-modal circuits in the brain cortex (Paperna and Malach 1991; Ibrahim et al. 2016). These horizontal connections can regulate cross-modal activation, as shown by elicited EPSCs in pyramidal and PV interneurons in VC layers 2/3 after light spot stimulation of AC axons (Ibrahim et al. 2016). In our material, the AC layer 2/3 of deaf animals are significantly more densely immunostained for GluA2/3 and for GAD67 (Fig. 11-2, 3). These findings highlighted the key role of horizontal feedbacks in over-activating the VC and maintaining the global inhibitory response of the AC. However, no significant changes in GABA receptor (*Gabra1* and *Gabbr2)* gene expression were detected by RT-qPCR.

GABAA ionotropic receptors have rapid rates of constitutive endocytosis (Kittler and Moss 2003; Kittler et al. 2005). In addition, GABAB metabotropic receptors present fast constitutive internalisation (Maier et al. 2010; Hannan et al. 2011), whose rate appears to depend on the GABAB-R2 subunit (Duthey et al. 2002; Margeta-Mitrovic et al. 2001; Hannan et al. 2011). After depolarising conditions by electric stimulation in cultured neurons, GABAA receptor subunit membrane expression increases without changes in the total quantity of protein, suggesting an increase in subunit trafficking without changes in transcription or synthesis (Rannals and Kapur 2011). Additionally, the activation of AMPA glutamate receptors in cortical cell cultures down-regulates GABAB receptors, as shown by western blot (Maier et al. 2010). Thus, based on the rates of internalisation and recycling of these receptors, increased inhibition (as shown by GAD analysis) may occur, with no relevant changes in the gene expression of GABA receptor subunits (as shown by RT-qPCR).

Immunocytochemistry for calcium-binding proteins (Celio 1986, 1990) and neuropeptides (Hendry et al. 1984) (PV, Calbindin/somatostatin and calretinin/vasointestinal peptide) identified three main phenotypes for GABA interneurons in the cortex (Kawaguchi and Kubota 1997). Furthermore, quantitative immunohistochemistry in the rat brain cortex has shown that approximately 40% of interneurons are immunoreactive to PV (Celio 1986; Lee et al. 2010; please see a review in Rudy et al. 2011). PV reactive interneurons correlate with the classical neuronal types of Basket and Chandelier cells (Kawaguchi and Kubota 1997). Thus, in our PV quantitative immunocytochemical analysis, we have explored approximately 40% of GABA interneurons of VC and AC and the two major short axon neuronal types involved in inhibitory regulation of cortical columns (Basket and Chandellier neurons—Fig. 11-3). After demonstrating, by depolarising current pulse and whole-cell recordings in rat brain slices, that PV cells have short-duration action potentials and fire repetitively without frequency adaptation, they were termed fast-spiking (FS) neurons (Kawaguchi 1993; Kawaguchi and Kubota 1993). Fast-spiking parvalbumin positive (FS-PV) interneurons closely regulate the discharge rate of pyramidal neurons, as clearly demonstrated by electrophysiology, more specifically by paired patch clamp recordings in brain slices of the cat VC (Tamás et al. 1997). A similarly tight regulation has been shown after in vivo patch-clamp recordings following optogenetic stimulation of Arch virus (Atallah et al. 2012). The FS of these interneurons is attributed to the high calcium affinity of the PV, which responds to increases in neuronal firing rate by buffering intracellular free Ca^2+^ concentrations (Chard et al. 1993). Consequently, significant increases in the number of FS-PV interneurons, which we have shown in the AC, would indicate that neuronal soma and dendrites are best detected by their higher immunoreactivity as a result of increased activation. Such an assertion is also indirectly supported by experiments made in rat hippocampal slices, which have shown that reduced Ca^2+^ inflow in FS-PV neurons by calcium chelators decreases the IPSC amplitude of pyramidal neurons (Bucurenciu et al. 2008). Considering the common phenotype of FS-PV and GABA neurons (Kawaguchi and Kubota 1997) and the known functional role of these interneurons, the increase in the immunoreactivity of FS-PV interneurons that we demonstrated could be related to an increase in GABA neurotransmission, thus further supporting, along with the GAD results, an increase in AC inhibition.

### Visual cortex plastic reaction

In congenital deafness (Neville et al. 1983) and after acquired hearing loss (Campbell and Sharma 2014), VEPs recordings have shown increases in N1 wave amplitudes, suggesting VC over-activation after long-term deafness in humans. In our experimental animal model, the mean of peak to peak N1 wave amplitude was 156.39% higher in deaf animals than in controls. N1 wave amplitude after VEP recordings reflects higher global electrical responses and VC over-activation after visual stimulation (Fig. 11, 1).

c-Fos is a marker of neuronal activity which is rapidly induced after neuronal depolarisation (Greenberg et al. 1986; Morgan and Curran 1988; Bartel et al. 1989; Joo et al. 2016). Accordingly, increased VC activation is confirmed by the overexpression of the activity-dependent early gene c-Fos (present results) and by significant increases in immunoreactive neurons in the VC (Pernia et al. 2017). Furthermore, a link between Arc/Arg3.1 expression and neuronal electrical activity has been demonstrated by the increase in Arc/Arg3.1 gene expression after high-frequency stimulation of the hippocampus (Lyford et al. 1995; Steward et al. 1998, 2015). Thus, indirect evidence at a cellular level of VC over-activation in our animal model also arises from significant increases in the expression of activity-dependent early genes c-Fos and Arc/Arg3.1.

The homeostatic plasticity of brain neuronal network regulation implies that any change in its activation may induce changes in receptor synthesis, trafficking and internalisation, which work in combination to adjust synaptic strength and/or neuronal membrane excitability (Turrigiano et al. 1998—for review see Pérez-Otaño and Ehlers 2005). The role of Arc/Arg3.1 in balancing excitability of neuronal networks by AMPA R regulation was analysed in mice hippocampal slices. The results showed that, after electric stimulation, Arc/Arg3.1 mediates the removal of GluA2/3 AMPA R subunits from the neuronal membrane and reduces the amplitude of EPSCs (Rial Verde et al. 2006). In our results, increases in activation and Arc/Arg3.1 expression in the VC did not coincide with any changes in GluA2 and GluA3 AMPA receptor subunits. Accordingly, considering the role of Arc/Arg3.1 in synaptic plasticity regulation, its increased expression suggests dynamic homeostatic compensation for VC neuronal network stabilisation after over-activation (Fig. 11b) (Rial Verde et al. 2006; Bramham et al. 2008; Wall and Corrêa 2018). Our results from all neuronal markers analysed in the VC indicated an increase in GAD67 immunostaining restricted to VC1, with no overall changes in gene expression in the VC. Because VC1 is the main recipient for visual primary thalamic inputs (Kharazia and Weinberg 1994), increased GAD67 immunostaining could be interpreted as a reactive response to changes in thalamocortical activation after deafness. Indeed, increased EPSC amplitude in VC1 layer 4 has been shown by recordings in deaf mice brain slices, in accordance with a potential over-activation of visual thalamic primary afferents after deafness (Petrus et al. 2014). However, our data thus also suggests that changes in GAD expression in the VC1 may result from imbalanced horizontal intermodal feedback between AC and VC (see below) (Fig. 11a).

## Concluding remarks and working hypothesis

Cross-modal balance for sensory processing between primary cortices results from a combination of thalamic driver activation, horizontal intermodal connections and intrinsic microcircuit elaboration of neuronal responses in the cortical columns.

VC overactivation (demonstrated by c-Fos analysis and VEPs recordings) could be generated by imbalanced horizontal interactions, as shown by changes in immunoreactivity specifically in layers 2/3. However, such imbalances do not equally affect both primary cortices, because GABA interneurons specifically increase in AC1 and GAD increases in primary and secondary ACs and in primary VC. Increases in gene expression and protein expression of AMPA receptors in layer 2/3 of the AC suggest an effort to compensate for changes in the activation of thalamic drivers. Overall, these results suggest that increased AC inhibition (GAD and PV-GABA neurons) is induced primarily by its plastic reorganisation after deafness and secondarily by the horizontal interaction from the over-activated VC (Fig. 11). Under these conditions, two homeostatic mechanisms actively work for dynamic compensation of the imbalanced bimodal relationship between AC and VC after deafness: (1) imbalanced horizontal cortical feedback (Fig. 11-a), and (2) Up-regulated Arc/Arg3.1 expression (which supports the hypothesis that a reactive homeostatic mechanism compensates for VC over-activation) (Fig. 11-b).

To establish a causal relationship between changes shown in both sensory cortices and thalamo-cortical regulation of VC and AC, paired recordings should be made in our animal model in future studies.

## Clinical implications

In prolonged deafness, a cross-modal reaction over-activates neighbouring sensory cortices (particularly VC), as shown in humans (Neville et al. 1983; Campbell and Sharma 2014). In this study, we demonstrated an unstable compensation of horizontal feedbacks between sensory cortices in our animal model. We have recently communicated that anodal continuous current stimulation allows restricted over-activation of the AC (Colmenárez-Raga et al. 2019). Restricted stimulation with anodal currents (activation) in the AC may be able to rebalance the cross-modal reaction, potentially improving cortical processing after cochlear implantation. Such an approach may induce a more efficient adaptation of cochlear implants in adults with long-term hearing loss and help to prevent cross-modal invasion in congenital deafness. A similar analysis to that presented herein, albeit in congenital deafness animal models, should be performed in future studies.

## Abbreviations

ABRs: Auditory brainstem recordings
AC: Auditory cortex
AC1: Primary auditory cortex
ACD: Dorsal auditory cortex
ACV: Ventral auditory cortex
AMPA Rs: AMPA receptors
dpl: Days post-lesion
FS: Fast-spiking
FS-PV: Fast-spiking parvalbumin
IA: Interaural
OD: Optical density
PB: Phosphate buffer
PtPD: Parietal postero-dorsal cortex
PtPR: Parietal postero-rostral cortex
PV: Parvalbumin
SSC: Somatosensory cortex
TBS-Tx: Tris-Buffered saline + 0.5% Triton X-100
VC: Visual cortex
VC1: Primary visual cortex
V2L: Secondary lateral visual cortex
V2M: Secondary medial visual cortex
V2MM: Secondary medio-medial visual cortex
V2ML: Secondary medio-lateral visual cortex
VEP: Visual evoked potential
